# Metallothionein genes: no association with Crohn's disease in a New Zealand population

**DOI:** 10.1186/1477-5751-11-8

**Published:** 2012-01-28

**Authors:** Angharad R Morgan, Alan G Fraser, Lynnette R Ferguson

**Affiliations:** 1 Discipline of Nutrition, FMHS, The University of Auckland, Auckland, New Zealand; 2 Nutrigenomics New Zealand, New Zealand; 3 Department of Medicine, FMHS, The University of Auckland, Auckland, New Zealand

**Keywords:** Crohn's disease, Metallothioneins, genetic association

## Abstract

Metallothioneins (MTs) are excellent candidate genes for Inflammatory Bowel Disease (IBD) and have previously been shown to have altered expression in both animal and human studies of IBD. This is the first study to examine genetic variants within the MT genes and aims to determine whether such genetic variants have an important role in this disease. 28 tag SNPs in genes MT1 (subtypes A, B, E, F, G, H, M, X), MT2, MT3 and MT4 were selected for genotyping in a well-characterized New Zealand dataset consisting of 406 patients with Crohn's Disease and 638 controls. We did not find any evidence of association for MT genetic variation with CD. The lack of association indicates that genetic variants in the MT genes do not play a significant role in predisposing to CD in the New Zealand population.

## Background

Inflammatory bowel disease (IBD) is a disorder characterised by chronic, relapsing inflammation of the gastrointestinal tract and is characterized by the presence of an increased level of reactive oxygen species in the mucosal intestinal tissue as well as oxidative DNA and protein damage, defective host-microbe interactions, immune cell infiltration, and disturbed T cell apoptosis. Metallothioneins (MTs) are able to affect all of these processes, making them good candidates for IBD. MTs are ubiquitous metal-binding proteins that have been highly conserved throughout evolution and are rapidly upregulated in response to an inflammatory stimulus. There are four main isoforms expressed in humans: MT1 (subtypes A, B, E, F, G, H, M, X), MT2, MT3 and MT4. These genes cluster together on a single locus on chromosome 16 (16q13) [1].

Support for the hypothesis of MTs playing an important role in the pathogenesis of IBD has come from various reports demonstrating altered MT expression in IBD. However these reports have been contradictory (for a summary see Waeytens *et al*., 2009 [2]) with some studies reporting MT upregulation in IBD [3,4] and others demonstrating a downregulation [5-11]. The inconsistencies between the various studies may be explained by differences in patients samples such as age, medication, disease activity, zinc status, as well as from where the tissue was sampled (ileum or colon).

Whilst there have been several studies investigating expression of MTs in IBD, there have been no studies published to date investigating genetic variants in MTs and IBD. The aim of this study is to identify SNPs across these genes and to genotype these in a well-characterised Caucasian New Zealand IBD dataset and to examine the results for evidence of genetic association between any of the MT genes and IBD.

## Methods

### Samples

A total of 1044 subjects from New Zealand were included in the study: 406 CD patients and 638 age and sex matched controls. All participants self-reported European ancestry.

Clinical records were analysed to confirm diagnosis, and IBD status was defined using standard diagnostic criteria [12]. Cases were phenotyped according to the Montreal Classification systems. Clinical characteristics of the CD patients are shown in table 1.

**Table 1:** Summary of clinical data of CD patients.

		CD
Gender	F	265 (65.6)
	M	139 (34.4)
Age at Diagnosis	< 17	46 (12.6)
	17 to 40	257 (70.2)
	40 <	63 (17.2)
CD Behaviour	Inflammatory	201 (55.1)
	Stricturing	118 (32.3)
	Penetrating	46 (12.6)
CD Location	Ileal	136 (37.2)
	Colonic	119 (32.5)
	Ileocolonic	111 (30.3)
Bowel resection	N	270 (66.7)
	Y	135 (33.3)
Other IBD family	N	330 (89.7)
	Y	38 (10.3)
EIM	N	301 (81.8)
	Y	67 (18.2)
Perianal disease	N	329 (85.7)
	Y	55 (14.3)

Participants consented to collection of peripheral blood or a buccal swab for DNA extraction and genotyping, and DNA was extracted from the blood/buccal samples using Qiagen's DNA extraction kit and following the manufacturer's instructions.

The study was conducted under ethical protocol MEC/04/12/011, authorised through the New Zealand Multi-Region Human Ethics Committee. All study subjects gave informed consent.

### Snp selection

Tag SNPs in genes MT1 (subtypes A, B, E, F, G, H, M, X), MT2, MT3 and MT4 were selected using Hapmap release 28, and the tagger functionality within Haploview with pairwise tagging to identify SNPs using an r^2 ^> 0.8 and a minor allele frequency > 5%. As a result 28 tag SNPs were selected for genotyping.

### Genotyping

Genotyping was performed with the MassARRAY and iPlex systems of the Sequenom genotyping platform (Sequenom, San Diego, CA), which uses the MALDI-TOF primer extension assay [13,14], according to manufacturers' recommendations.

Assays were optimized in 24 samples consisting of 20 reference Centre d'Etude du Polymorphisme Humain (CEPH) samples and 4 blanks.

All sample plates contained cases, controls, blanks, CEPH and duplicate samples. Quality control measures included independent double genotyping and, where available, comparison of our CEPH genotypes to those in the Hapmap database http://www.hapmap.org.

### Statistical analysis

SNPs were tested for deviation from HWE in both cases and controls using a chi-square goodness-of-fit test. To determine if there were differences between cases and controls, genotype and allele frequencies for each SNP were analyzed by 2 × 3 and 2 × 2 Chi-square tables respectively

Genotype and phenotype associations were assessed by comparing allele frequencies between controls and patient subgroups defined using the clinical characteristics. These analyses were carried out using R (R: A language and environment for statistical computing, R Foundation for Statistical Computing, Vienna, Austria. ISBN 3-900051-07-0, URL http://www.R-project.org/) and SAS (V9.1 SAS Institute., Cary, NC, USA).

To determine linkage disequilibrium (LD) between SNPs and to define haplotype blocks, we uploaded our data into Haploview [15]. Haplotype blocks were defined using the default algorithm which uses confidence intervals [16]. Haplotype analysis was carried using HAPLO.SCORE in R to test for association of these haplotypes with CD.

For all analyses we considered a p values less than 0.05 to indicate statistical significance.

The false discovery rate (FDR) was used to correct for multiple testing [17,18].

## Results and discussion

Three SNPs did not fit into the Sequenom multiplexes: rs1827210 (MT1M), rs11076161 (MT1A), rs2298846 (MT1G). Two SNPs failed to be successfully genotyped: rs1827208 (MT1M) and rs12448654 (MT1G). One SNP was out of HWE in the control samples and so was excluded from further analysis: rs12051311 (MT1B). Thus there were 22 SNPs with genotype data for analysis. Genotype and allele counts/frequencies and p-values are shown in table 2. One SNP rs4784708 (MT1H) was found to be associated with CD (genotypic p = 0.0197, allelic p = 0.016) but after applying multiple testing correction using FDR the result is no longer statistically significant. Thus we can conclude that individual SNPs in the MT genes are not associated with CD.

**Table 2:** Genotype and allele counts (and frequencies) in CD patients and in controls

SNP	Location	gene		CASE	CONTROL	p		CASE	CONTROL	p
rs182221	56, 599, 221	MT4	G/G	262 (0.66)	398 (0.64)	0.87	G	646 (0.81)	995 (0.80)	0.72
			C/G	122 (0.31)	199 (0.32)		C	154 (0.19)	247 (0.20)	
			C/C	16 (0.04)	24 (0.04)					
rs666636	56, 601, 720	MT4	G/G	386 (0.95)	599 (0.95)	0.70*	G	791 (0.98)	1229 (0.97)	0.55
			A/G	19 (0.05)	31 (0.05)		A	19 (0.02)	35 (0.03)	
			A/A	0 (0.00)	2 (0.00)					
rs666647	56, 601, 722	MT4	C/C	386 (0.95)	605 (0.95)	0.73*	C	791 (0.98)	1241 (0.97)	0.58
			C/T	19 (0.05)	31 (0.05)		T	19 (0.02)	35 (0.03)	
			T/T	0 (0.00)	2 (0.00)					
rs669293	56, 602, 352	MT4	T/T	293 (0.73)	428 (0.67)	0.16	T	686 (0.85)	1039 (0.82)	0.051
			C/T	100 (0.25)	283 (0.29)		C	120 (0.15)	231 (0.18)	
			C/C	10 (0.02)	24 (0.04)					
rs762604	56, 602, 671	MT4	C/C	183 (0.45)	302 (0.48)	0.18	C	538 (0.67)	878 (0.70)	0.15
			C/T	172 (0.43)	274 (0.44)		T	268 (0.33)	380 (0.30)	
			T/T	48 (0.12)	53 (0.08)					
rs11643815	56, 602, 798	MT4	G/G	307 (0.76)	482 (0.76)	0.53	G	700 (0.86)	1102 (0.87)	0.61
			A/G	86 (0.21)	138 (0.22)		A	110 (0.14)	162 (0.13)	
			A/A	12 (0.03)	12 (0.02)					
rs11644094	56, 624, 079	MT3	A/A	134 (0.34)	220 (0.35)	0.90	A	460 (0.58)	736 (0.58)	0.79
			A/G	192 (0.48)	295 (0.47)		G	338 (0.42)	527 (0.42)	
			G/G	73 (0.18)	116 (0.18)					
rs10636	56, 643, 343	MT2A	G/G	228 (0.57)	358 (0.57)	0.66	G	598 (0.74)	946 (0.75)	0.67
			C/G	142 (0.35)	230 (0.37)		C	206 (0.26)	312 (0.25)	
			C/C	32 (0.08)	41 (0.07)					
rs2070836	56, 660, 120	MT1E	G/G	346 (0.85)	530 (0.84)	0.56	G	746 (0.92)	1156 (0.92)	0.69
			C/G	54 (0.13)	96 (0.15)		C	64 (0.08)	106 (0.08)	
			C/C	5 (0.01)	5 (0.01)					
rs708274	56, 660, 906	MT1E	G/G	310 (0.78)	482 (0.77)	0.22	G	701 (0.88)	1100 (0.88)	0.80
			G/T	81 (0.20)	136 (0.22)		T	95 (0.12)	144 (0.12)	
			T/T	7 (0.02)	4 (0.01)					
rs2270836	56, 667, 614	MT1M	C/C	169 (0.42)	231 (0.37)	0.19	C	516 (0.64)	764 (0.61)	0.083
			C/T	178 (0.44)	302 (0.48)		T	286 (0.36)	498 (0.39)	
			T/T	54 (0.13)	98 (0.16)					
rs9936741	56, 667, 785	MT1M	T/T	387 (0.96)	610 (0.95)	0.94*	T	792 (0.98)	1249 (0.98)	0.94
			C/T	18 (0.04)	29 (0.05)		C	18 (0.02)	29 (0.02)	
			C/C	0 (0.00)	0 (0.00)					
rs7190725	56, 673, 290	MT1A	G/G	308 (0.76)	483 (0.76)	0.99	G	706 (0.87)	1109 (0.87)	0.94
			G/T	90 (0.22)	143 (0.22)		T	104 (0.13)	165 (0.13)	
			T/T	7 (0.02)	11 (0.02)					
rs964372	56, 686, 030	MT1B	G/G	317 (0.78)	514 (0.81)	0.65	G	714 (0.88)	1142 (0.89)	0.34
			C/G	80 (0.20)	114 (0.18)		C	96 (0.12)	134 (0.11)	
			C/C	8 (0.02)	10 (0.02)					
rs2070839	56, 686, 242	MT1B	C/C	152 (0.38)	256 (0.40)	0.17	C	503 (0.63)	792 (0.62)	0.89
			C/T	199 (0.50)	280 (0.44)		T	301 (0.37)	480 (0.38)	
			T/T	51 (0.13)	100 (0.16)					
rs2291956	56, 692, 218	MT1F	C/C	308 (0.76)	481 (0.76)	0.97	C	705(0.87)	1104 (0.87)	0.97
			C/T	89 (0.22)	142 (0.22)		T	103 (0.13)	162 (0.13)	
			T/T	7 (0.02)	10 (0.02)					
rs2298847	56, 701, 294	MT1G	T/T	310 (0.77)	495 (0.79)	0.28	T	709 (0.88)	1107 (0.89)	0.58
			A/T	89 (0.22)	117 (0.19)		A	99 (0.12)	143 (0.11)	
			A/A	5 (0.01)	13 (0.02)					
rs4784708	56, 703, 914	MT1H	G/G	376 (0.94)	565 (0.89)	0.0197*	G	778 (0.97)	1196 (0.94)	**0.016**
			C/G	26 (0.06)	66 (0.10)		C	26 (0.03)	70 (0.06)	
			C/C	0 (0.00)	2 (0.00)					
rs2062545	56, 704, 125	MT1H	A/A	364 (0.90)	580 (0.91)	0.43*	A	768 (0.95)	1214 (0.96)	0.42
			A/G	40 (0.10)	54 (0.09)		G	42 (0.05)	56 (0.04)	
			G/G	1 (0.00)	1 (0.00)					
rs2062546	56, 704, 217	MT1H	T/T	353 (0.87)	566 (0.89)	0.35*	T	757 (0.94)	1200 (0.95)	0.36
			A/T	51 (0.13)	68 (0.11)		A	51 (0.06)	68 (0.05)	
			A/A	0 (0.00)	0 (0.00)					
rs2301234	56, 716, 982	MT1X	G/G	173 (0.43)	248 (0.39)	0.41	G	517 (0.64)	791 (0.62)	0.35
			G/T	171 (0.43)	395 (0.46)		T	287 (0.36)	479 (0.38)	
			T/T	58 (0.14)	92 (0.14)					

*p value calculated from comparing rare homozygote + heterozygote group against common homozygote group

Calculations of statistical power using PS 2.1.31 [19] show that using our case-control sample set although we have good power to detect an odds ratio of 1.3 for common SNPs (for example for a SNP with MAF 0.42 (e.g., rs11644094) we have 86.5% power) the power is considerably lower for SNPs with low MAF. For example for a SNP with MAF of 0.02 (e.g., rs9936741) we have 16.1% power. Thus, an association of metallothionein genes with CD cannot be entirely ruled out as it may simply be that we do not have the power in our study to detect such an association. A replication study in a larger sample set is encouraged.

**Figure 1:**
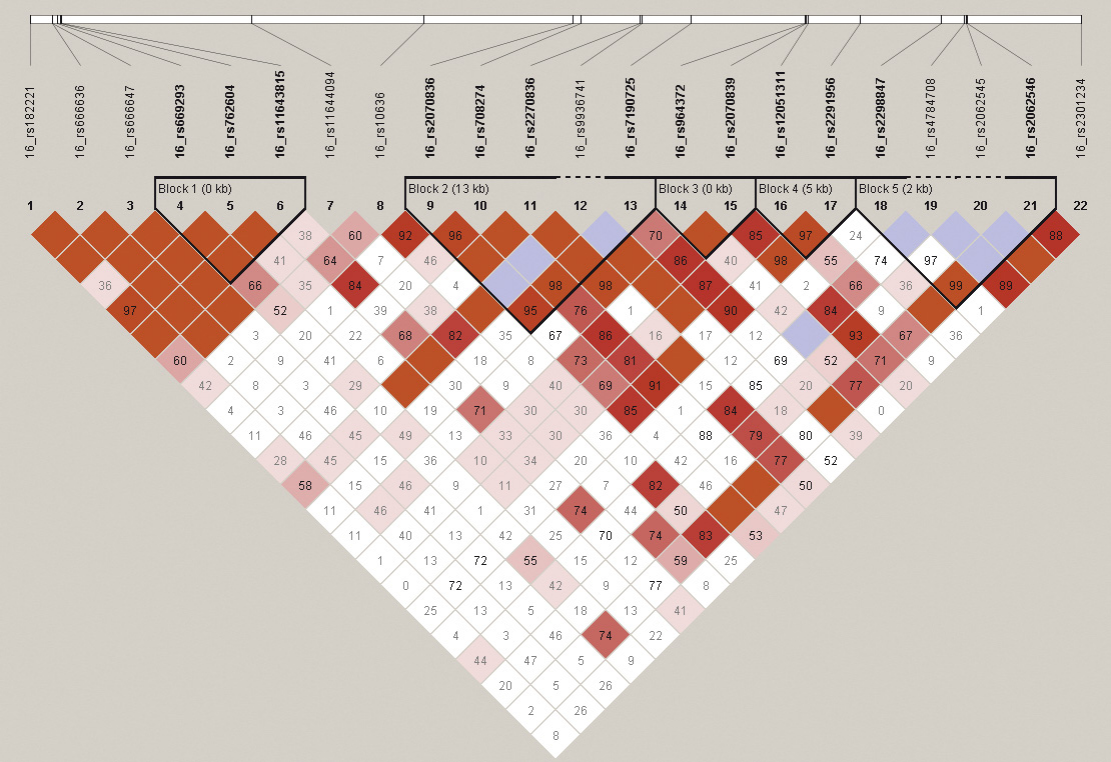
LD plot

There are 5 haplotype blocks in this region (Figure 1) but examining the different haplotypes we did not find any associations with CD (data not shown but available on request).

We also undertook a phenotype analysis and after multiple testing corrections none of the SNPs were associated with any of the different clinical characteristics of CD (data not shown but available on request).

This is the first study to investigate genetic association with MT SNPs and IBD. In summary although MT genes are good candidates for CD, the present study failed to detect any significant associations. The lack of association indicates that genetic variants in the MT genes do not play a significant role in predisposing to CD in the New Zealand population.

## Competing interests

The authors declare that they have no competing interests.

## Authors' contributions

ARM designed the study, performed the genotyping and QC, analysis and interpretation of data. She was primary author of the manuscript. AGF participated in participant recruitment and collection of clinical information from the patients. LRF was responsible for obtaining funding. All authors read and approved the final manuscript.
